# A Preliminary Study on Exploring a potential Ultrasound Method for Predicting Cervical Cancer

**DOI:** 10.7150/jca.60413

**Published:** 2022-01-01

**Authors:** Qiuqing Zheng, Chunyi Lin, Dong Xu, Huicheng Zhao, Mei Song, Di Ou, Le Shi

**Affiliations:** 1 Department of ultrasound, The Cancer Hospital of the University of Chinese Academy of Sciences (Zhejiang Cancer Hospital), Institute of Basic Medicine and Cancer (IBMC), Chinese Academy of Sciences, Hangzhou, Zhejiang, China.; 2 Institute of electronics and information, South China University of technology, Guangzhou, China.

**Keywords:** tissue typing, ultrasound, radio frequency time series signal, cervical cancer

## Abstract

**Background:** The level of cervical cancer screening in underdeveloped countries is far behind that of developed countries mostly because current cervical cancer screening methods are difficult to implement in underdeveloped countries. The use of non-invasive, repeatable, and low-cost ultrasound needs to be accessed.

**Methods:** The Canadian Sonix TOUCH ultrasound system and transvaginal ultrasound probe were used to record ultrasound radio frequency (RF) signals from cervical tissues of 69 patients with cervical cancer and 37 healthy women. The self-compiled RF time series signal analysis software was used to extract 3 different dimensions of parameters of the region of interest (ROI), including time domain, frequency domain, and fractal dimension (FD). Fourteen spectrum characteristic parameters were extracted, of which structure function method FD (SFD) and Higuchi FD belonged to FD parameters; slope, intercept, midbandfit, S1, S2, S3, and S4 were frequency domain parameters; and fuzzy entropy, kurtosis, peak, cross zero count, and cross zero standard deviation (Std) were time domain parameters.

**Results:** The average values of the five time-domain characteristic parameters of cervical cancer tissues were smaller than those of normal cervical tissues (fuzzy entropy: 1.70±0.29 vs. 1.83±0.20; kurtosis: 0.347±0.03 vs. 0.350±0.02; peak: 1989.9±166.8 vs. 2024.69±187.5; cross zero count: 3.77±0.31 vs. 3.81±0.29; cross zero Std: 1.26±0.17 vs. 1.33±0.14), although the differences were not statistically significant (*P =* 0.130, 0.326, 0.618, 0.442, and 0.204, respectively). The average values ​​of the two FD characteristic parameters and the seven frequency domain characteristic parameters of cervical cancer tissues were larger than those of normal tissues (SFD: 1.84±0.28 vs. 1.46±0.39; Higuchi FD: 1.71±0.30 vs. 1.28±0.30; slope: -0.32±0.08 vs. -0.26±0.05; intercept: 0.48±0.02 vs. 0.46±0.02; midbandfit: 0.35±0.03 vs. 0.33±0.03; S1: 15.66±1.01 vs. 13.57±1.69; S2: 10.12±0.69 vs. 9.32±1.27; S3: 9.44±1.12 vs. 8.66±1.09; S4: 7.67±1.01 vs. 6.43±0.65), and the differences were statistically significant (*P* < 0.05). No effective parameters were found to identify cervical squamous cell carcinoma tissues with different levels of differentiation (*P* > 0.05).

**Conclusion:** Quantitative analysis of RF time series signals based on ultrasound RF flow is expected to become a simple and non-invasive imaging method for cervical cancer diagnosis. However, whether it can be applied to the identification of early small cervical cancer lesions remains to be determined.

## Introduction

In the past decades, the incidence and death rates of cervical cancer have decreased dramatically in developed countries partly due to advances in screening methods and human papillomavirus (HPV) vaccines 1-3. However, the situation in underdeveloped countries is different. HPV vaccines are scarce in underdeveloped countries, and although high-quality screening can compensate for this shortcoming, cervical cancer screening in underdeveloped countries remains unsatisfactory. The screening process for cervical cancer, which includes cervical cytology, HPV tests, colposcopy, and cervical biopsy, is complex, time-consuming, and psychologically taxing 4. In underdeveloped countries, the distribution of medical resources is unbalanced. In economically backward areas and primary hospitals, the promotion and application of standard cervical cancer screening procedures are limited by the lack of equipment and professional technicians 5. The occurrence of cervical cancer is related to HPV infection. Cervical cytological screening, high-risk HPV detection, and combined screening are effective clinical detection and screening methods. Among them, liquid-based thin-layer cytology (TCT) is the first choice for cervical cancer screening, as the accuracy of examination can reach 100% in theory 6. However, medical resources are limited in economically backward areas, and gynecologists and cytologists are not well qualified for the TCT, resulting in high false negative rates and false positive rates of cervical screening. Therefore, decreasing the incidence and mortality of cervical cancer in underdeveloped countries remains an unmet goal 2, 3, 7, 8.

Developing simpler, more homogeneous, and non-invasive imaging methods is important. Ultrasonography is a flexible green imaging method associated with several advantages, such as low-cost equipment, easy to carry, no radiation, no pain, and repeatable operation. Ultrasonography allows visualization of the shape, internal structure, boundary, and blood flow of the lesion to help distinguish benign from malignant tumors. As a first-line imaging technology in various clinical fields, ultrasonography is widely used and applied to tumor screening worldwide. Ultrasound has been used for screening of liver cancer, thyroid cancer, and breast cancer among others. A national multicenter breast screening study led by Shen et al. revealed that the sensitivity and accuracy of ultrasonography are superior to those of radiography for breast screening 9. The American College of Radiology Imaging Network breast screening study 6666 project results showed that ultrasound was more effective for screening breast invasive carcinoma than X-ray 10. However, traditional imaging methods including ultrasound are not effective for detecting early cervical cancer lesions 11-13. Although imaging is included in the 2018 cervical cancer National Comprehensive Cancer Network guidelines as the staging and treatment guidance standard, it is only applicable to the middle and advanced stages of the disease 7, 14.

Similarly, imaging methods have limited efficacy for the early diagnosis of prostate cancer 15. Researchers have attempted to identify prostate cancer tissue by ultrasonic tissue characterization. Ultrasound RF time series analysis was considered to be one of the most effective ultrasonic tissue characterization methods, which can find subtle differences between tissues 16, 17. Ultrasound RF time series signals have even been used to assess the subtle changes of tumors at the beginning of chemotherapy 18, 19. Inspired by those, we used ultrasound RF time series analysis to identify cervical cancer tissues in this study. Because of the limited experience and because there is no precedent for the use of this method in the identification of cervical cancer tissues, we screened cases of advanced cervical cancer into the study, which were quite different from normal cervical tissues and could be visualized by traditional ultrasound.

## Materials and Methods

### Study population

The study was conducted between March 2015 and January 2016 at Zhejiang Cancer Hospital. The study population consisted of 69 cervical carcinoma patients and 37 health controls. All enrolled cervical cancer patients were pathologically confirmed squamous cell carcinoma of the uterine cervix with nodules visualized by transvaginal ultrasound. Patients had received previous treatment, had recurrent neoplasms or had a secondary primary malignancy were excluded. 37 healthy controls were all healthy women who came to our hospital for physical examination. They had no previous cervical disease and had not undergone cervical surgery before. All subjects were able to tolerate transvaginal ultrasound and signed informed consent before enrollment. The study was approved by the Clinical Research Ethics Committee of the Zhejiang Cancer Hospital.

### Ultrasound data collection

RF ultrasound data were collected using a Sonix TOUCH (Ultrasonix Medical Corp., Richmond, BC, Canada) ultrasound machine capable of recording raw RF data. A transvaginal ultrasound probe was used with the central frequency set to 6 MHz and a depth of 10 cm. To collect the RF time series signals, the sonographer performed a preliminary ultrasound scan to locate the cancerous nodule and then held the probe steady for 20 s while a computer program stored the RF data into the memory.

### Parameter extraction

**Figure 1:**
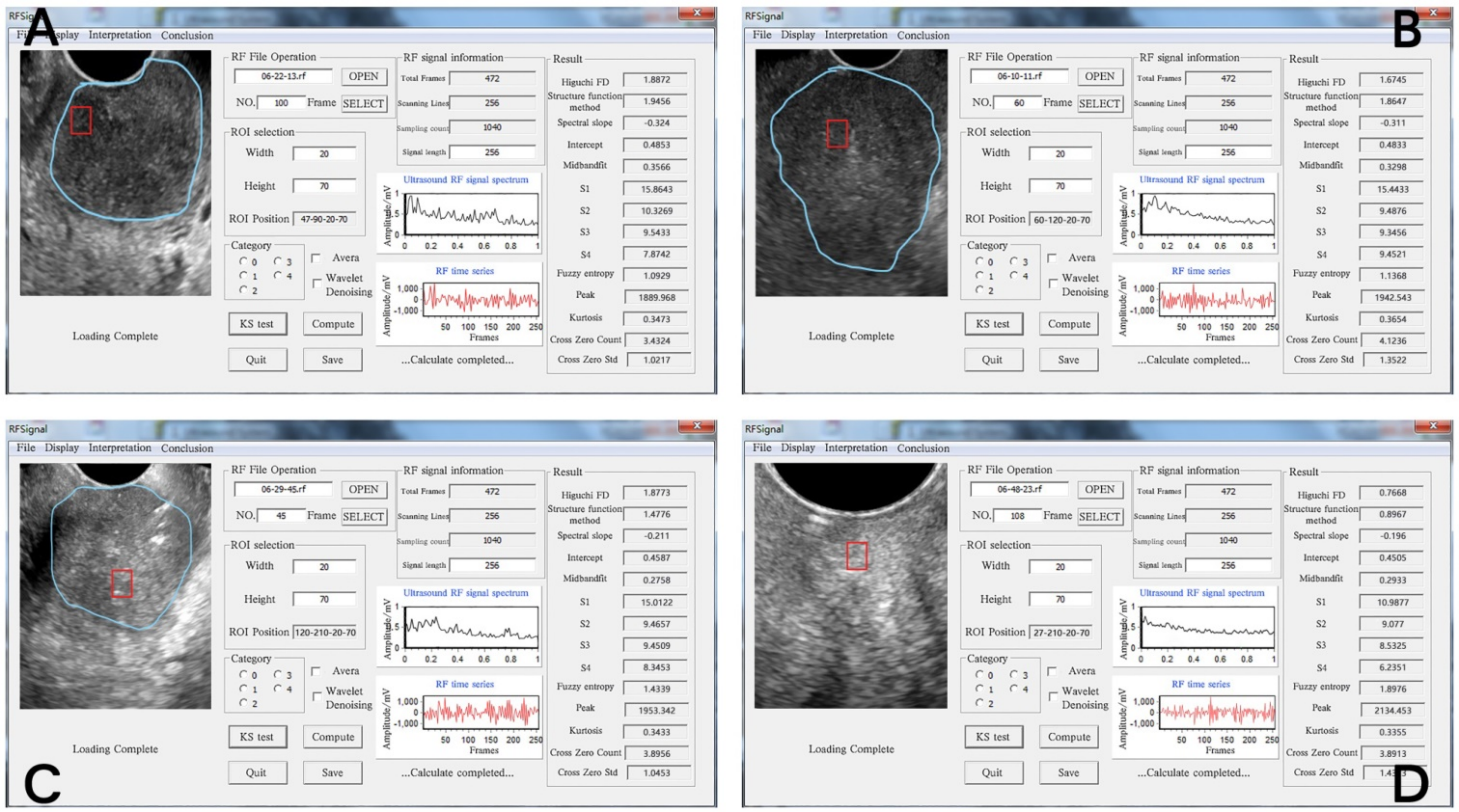
**Operation interface for extracting RF time series parameters from cervical tissue.** The four images represent the diagrams of the extraction operation interface of the RF time series parameters of different pathological types of cervical tissue. The ultrasound image of different cervical tissues is on the left of each interface; the blue line outlines the cervical cancer lesion area, and the red line outlines the ROI. The middle area of the interface is the respective RF time series spectrogram. The respective 14 RF time series parameter values are shown on the right side of the interface diagram. The pathological types of the four ROIs were as follows: **(A)** poorly differentiated cervical squamous cell carcinoma, **(B)** middle-low differentiated cervical squamous cell carcinoma, **(C)** middle differentiated cervical squamous cell carcinoma, **(D)** normal cervix with a cytology negative for intraepithelial lesion or malignancy.

In the current study, the sonographer outlined the cancerous nodule region in the B-mode ultrasound image to allow the programmer to select the cancerous ROI accurately. The ROI size was 70 × 20 mm. We used the first 256 frames of the back scattering echo RF signal to form the 1400 RF time series, which was used to extract the tissue characterizing features. The RF time series signal analysis software developed by the team was used for quantitative analysis of the ROI 13. The FD, frequency domain, and time domain features of the RF time series were extracted, including 14 parameters in total (Figure 1). The calculation methods for extracting all parameters are detailed in Supplementary. Among them, SFD and Higuchi FD belong to FD parameters; slope, intercept, midbandfit, S1, S2, S3, and S4 belong to frequency domain parameters; and fuzzy entropy, kurtosis, peak value, cross zero count, and cross zero Std are time domain parameters.

### Results analysis

The classification results were compared with the pathologic results, which were used as the gold standard. SPSS version 22.00 statistical software was used for statistical analysis. All research data in this study were normally distributed and had uniform variance; therefore, the T test was used to compare the RF time series parameters of normal and malignant cervical tissues, and one-way analysis of variance (ANOVA) was used to compare RF time series parameters among cervical cancer tissues with different degrees of differentiation. All results were considered statistically significant at *P* < 0.05.

## Results

### Pathological results

**Figure 2:**
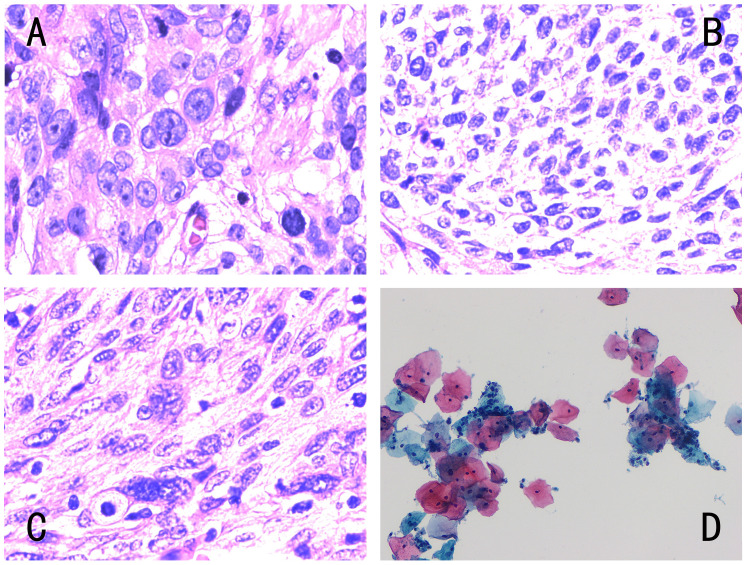
** Different types of cervical pathology.** The four images corresponded to Fig. 1, and the pathological results were as follows: **(A)** histopathology of low differentiated cervical squamous cell carcinoma, **(B)** histopathology of middle-low differentiated cervical squamous cell carcinoma, **(C)** histopathology of middle differentiated cervical squamous cell carcinoma, **(D)** cytology negative for intraepithelial lesion or malignancy. Histology was diagnosed by hematoxylin-eosin (H&E) staining (magnification, x400) and cytology by Papanicolaou staining (magnification, x400).

All cases analyzed had clear pathological results (Table 1) (Figure 2). The 69 cases of cervical squamous cell carcinoma were confirmed by histopathology. Among them, there were 24 cases of moderately differentiated carcinoma, 17 cases of moderate-low differentiated carcinoma, and 28 cases of poorly differentiated carcinoma. The 37 women with a normal cervix included in the control group underwent cervical cytology, and the results showed “negative for intraepithelial lesion or malignancy (NILM)”. Because none of the 37 healthy women in the control group had a clinical symptom and the cytopathology from the cervical screening in our hospital was normal, no further histopathological tests were performed. Therefore, histopathology was not available.

### Correlation between ultrasound RF time series features and pathological results

A total of 14 RF time series parameters were obtained by quantitative analysis of time series signals based on ultrasonic RF flow, all of which showed normal distribution. The mean values of FD and frequency domain parameters of cervical cancer (SFD: 1.84±0.28, Higuchi FD: 1.71±0.30, slope: -0.32±0.08, intercept: 0.48±0.02, midbandfit: 0.35±0.03, S1: 15.66±1.01, S2: 10.12±0.69, S3: 9.44±1.12, S4: 7.67±1.01) were higher than those of normal cervical tissues (SFD: 1.46±0.39, Higuchi FD: 1.28±0.30, Slope: -0.26±0.05, intercept: 0.46±0.02, midbandfit: 0.33±0.03, S1: 13.57±1.69, S2: 9.32±1.27, S3: 8.66±1.09, S4: 6.43±0.65), and the difference was statistically significant (*P* < 0.05), On the contrary, the mean values of time domain characteristic parameters of cervical cancer (fuzzy entropy:1.70±0.29, kurtosis: 0.347±0.03, peak: 1989.9±166.8, cross zero count: 3.77±0.31, cross zero Std: 1.26±0.17) were smaller than those of normal cervical tissues, but the differences were not statistically significant (*P* = 0.130, 0.326, 0.618, 0.442, and 0.204, respectively) (Table 2).

### Comparison of RF time series parameters among cervical squamous cell carcinoma tissues with different degrees of differentiation

Higher mean values of slope, intercept, S2, and S4 were correlated with a worse degree of differentiation (Table 3). Although the above parameters showed changes consistent with the changes in tissue differentiation, no statistically significant differences were found among three groups with different degrees of differentiation (*P* > 0.05).

## Discussion

Cervical cancer is the fourth most common cancer among women in the world, and its prognosis and survival are closely related to the stage of the disease 1. The 5-year survival rate for localized cervical cancer is 91.5%, while for late metastases it drops to 16.5% 20. In developed countries, due to the popularity of HPV vaccine and cervical cancer screening, encouraging results have been shown in reducing the morbidity and mortality of cervical cancer patients. However, in underdeveloped countries due to the lack of medical resources, women cannot get effective early diagnosis. Most of them are already in the advanced stage when they are discovered, often accompanied by uncomfortable clinical symptoms such as abnormal vaginal bleeding. This makes standard treatment strategies ineffective, which not only affecting prognosis, but also disturbing with their normal lives 2, 4, 6. Therefore, it is very important to make it easier for women in underdeveloped countries to get early detection of cervical cancer.

Ultrasound examination is a convenient and effective method for clinical diagnosis and treatment guidance in many diseases because it does not expose the patient to radiation, it is non-invasive, can be easily repeated, and it is cost-effective. Traditional gray-scale ultrasound imaging uses the ultrasound probe to receive the envelope amplitude of the RF echo signal. This signal processing ignores some information in the original RF signal stream, such as the frequency spectrum, phase, and the interaction between soft tissue and ultrasound. It can distinguish tissue structures with a diameter larger than the wavelength, and cannot evaluate the microstructure characteristics of tissues with a diameter smaller than the wavelength 21. Therefore, ultrasound imaging has several shortcomings in clinical practice, such as its inability to identify certain tumor lesions, especially early small lesions. In recent years, many studies have investigated methods to extract the information related to the tissue microstructure in the original ultrasound RF signal to characterize the tissue microstructure. Among them, ultrasonic RF time series signal analysis is a widely used and reliable method. The early application of this technology in the field of cancer was limited to the identification of tumor tissues, such as prostate cancer and breast cancer 16, 17, 22. Later studies used the same organizational characterization method to observe the response to treatment. Imani et al. used this method to distinguish ablated from non-ablated liver tissues 23, whereas Li et al. used the method to identify chemotherapy responders and non-responders in preclinical breast cancer models 19. These studies demonstrated that the RF time series analysis method is capable of characterizing the subtle differences between different tissues.

In this study, cervical cancer and normal cervical tissues were distinguished by analyzing the ultrasound RF time series signals of the microstructure of the lesion. We extracted features from three dimensions including time domain, frequency domain, and FD, and 14 parameters were extracted in total. Among them, the average values of the five time domain parameters of cervical cancer were smaller than those of normal tissues. The larger the value of the time domain parameters, the more uniform the tissue distribution 24, 25. However, the differences were not statistically significant. Studies show that when performing time domain analysis of RF signals, finding the same time domain parameters of some signals does not indicate that the signals are exactly the same. The signal not only changes with time, but it is also related to information such as frequency and phase. Time domain functions can be transformed into frequency domain functions by Fourier or Laplace transformation 26-28. According to this method, we further extracted nine parameters of frequency domain and FD. The mean values of these nine parameters in cervical cancer and normal cervical tissues were significantly different, which is consistent with previous studies. Moradi et al. found that six frequency domain parameters including slope, intercept, S1-4, and FD have good sensitivity and specificity for identifying prostate cancer 16. Analysis of six different animal tissues showed that the six frequency domain parameters and FD have high accuracy, and FD has the highest accuracy (74%-90%) 29. Uniyal et al. reclassified suspected breast cancer tissues, and found that frequency domain and FD parameters played an important role; the AUC values were estimated at 0.82 (95% CI: 0.63-1.00) using support vector machines and 0.69 (95% CI: 0.47-0.91) using Random Forests 22.

In this study, we further compared the ultrasound RF time series signal parameters of cervical squamous cell carcinoma tissues with different levels of differentiation. Because we screened and enrolled all cases of cervical cancer that were visualized by grayscale ultrasound, they were all in the middle and late stages of the tumor and poorly differentiated; therefore, no cases of well-differentiated cancer were detected in this study. Among 28 cases of poorly differentiated cervical squamous cell carcinoma, 17 cases of moderate-poorly differentiated squamous cell carcinoma, and 24 cases of moderately differentiated squamous cell carcinoma tissues, the four parameters slope, intercept, S2, and S4 varied in a pattern consistent with the changes in tissue microstructure. However, the differences were not statistically significant (*P*>0.05). This may be related to the small number of cases enrolled and the small differences between the tissues of the enrolled cases.

Frequency domain and FD of ultrasonic RF time series signals are parameters that reflect the complexity, roughness, and irregularity of the tissue surface, which may overlap with traditional gray-scale ultrasound imaging in identifying tissues. In this study, the use of ultrasonic RF time series signals to identify cervical cancer tissue was a preliminary and simple study. The objects selected were all cervical cancer tissues that can be visualized by traditional gray-scale ultrasound. Whether this method is suitable for the early diagnosis of cervical cancer remains unknown, and further in-depth studies will be performed in the future, such as using small cervical cancer lesions as ROI. These lesions can only be identified by contrast ultrasound, but not gray-scale ultrasound. We hope that additional efforts will result in the use of this technology to identify early cervical cancer, and ultimately assist in the screening of cervical cancer.

## Conclusion

Quantitative analysis of ultrasound RF time series parameters can be used for the identification of cervical cancer; however, its feasibility for the identification of cervical cancer tissues with different levels of differentiation has not been demonstrated, and whether it can be applied to early cervical cancer tissues that cannot be identified by gray-scale ultrasound remains unknown. This preliminary study showed that this method can be applied to the identification of cervical cancer, thereby providing a potential new ultrasound-based method for the diagnosis of cervical cancer. Additional studies are necessary to promote the application of ultrasound to the early diagnosis and screening of cervical cancer in future.

## Supplementary Material

Supplementary information.

## Figures and Tables

**Table 1:** Pathological Classifications of 106 subjects

Classification	Number of cases
Low differentiated cervical SCC	28
Middle-low differentiated cervical SCC	17
Middle differentiated cervical SCC	24
NILM	37

Abbreviations: SCC, squamous cell carcinoma; NILM, negative for intraepithelial lesion or malignancy.

**Table 2:** Comparison of Ultrasound RF Time Series Parameters between cervical squamous cell carcinoma and normal cervical tissues

Parameter	Average value		*P* value
	Tumor	Normal	
Higuchi FD	1.71±0.30	1.28±0.30	<0.001*
SFD	1.84±0.28	1.46±0.39	<0.001*
Slope	-0.32±0.08	-0.26±0.05	<0.001*
Intercept	0.48±0.02	0.46±0.02	<0.001*
Midbandfit	0.35±0.03	0.33±0.03	0.022*
S1	15.66±1.01	13.57±1.69	<0.001*
S2	10.12±0.69	9.32±1.27	0.001*
S3	9.44±1.12	8.66±1.09	<0.001*
S4	7.67±1.01	6.43±0.65	<0.001*
Fuzzy entropy	1.70±0.29	1.83±0.20	0.130
Peak	1989.9±166.8	2024.69±187.5	0.326
Kurtosis	0.347±0.03	0.350±0.02	0.618
Cross Zero Count	3.77±0.31	3.81±0.29	0.442
Cross Zero Std	1.26±0.17	1.33±0.14	0.204

*P* < 0.05 indicates statistical significance; Abbreviations: Higuchi FD, Higuchi fractal dimension; SFD, Structure function method fractal dimension; Cross Zero Std, Cross Zero Standard deviation.

**Table 3:** Comparison of Ultrasound RF Time Series Parameters among Different Differentiated Cervical Squamous Cell Carcinoma tissues

Parameter	Cervical squamous cell carcinoma			*F* value	*P* value
	Low differentiated carcinoma	Middle-low differentiated carcinoma	Middle differentiated carcinoma		
Higuchi FD	1.861±0.31	1.87±0.39	1.796±0.19	0.376	0.688
SFD	1.681±0.32	1.707±0.39	1.754±0.939	0.468	0.628
Slope	-0.325±0.03	-0.320±0.08	-0.302±0.09	0.577	0.564
Intercept	0.483±0.18	0.481±0.19	0.478±0.18	0.369	0.693
Midbandfit	0.353±0.03	0.360±0.03	0.342±0.03	1.942	0.151
S1	15.621±0.99	15.837±1.19	15.587±0.93	0.337	0.715
S2	10.246±0.63	10.183±0.49	9.589±0.84	1.129	0.33
S3	9.689±0.73	8.919±1.34	9.52±1.25	2.694	0.075
S4	7.747±0.87	7.623±1.08	7.602±1.01	0.148	0.862
Fuzzy entropy	1.763±0.23	1.625±0.29	1.616±0.32	1.817	0.171
Peak	1990.1±199.8	2065.6±195.8	1935.9±185.3	2.602	0.074
Kurtosis	0.352±0.03	0.345±0.03	0.342±0.03	0.62	0.541
Cross Zero Count	3.720±0.33	3.739±0.31	3.822±0.30	0.808	0.45
Cross Zero Std	1.279±0.17	1.2321±0.18	1.253±0.17	0.418	0.66

*P* < 0.05 indicates statistical significance; Abbreviations: Higuchi FD, Higuchi fractal dimension; SFD, Structure function method fractal dimension; Cross Zero Std, Cross Zero Standard deviation.
