# Supplementary material for: A Preliminary Study on Exploring a potential Ultrasound Method for Predicting Cervical Cancer
Source: J Cancer. 2022 Jan 1;13(3):793–9. doi: 10.7150/jca.60413 (PMC8824887; doi:10.7150/jca.60413)
Supplement: Supplementary file 1 — Supplementary information. [file jcav13p0793s1.pdf]

The calculation formulas for extracting all RF time series parameters are as follows:

1. FD:
   (1) Higuchi FD:
      a. Suppose the time series of N points is $\{x(i): 1 \le i \le N\}$
      b. Construct a new time series from the given time series: $x_k^m = \{x(m + i * k)\}, m = 1,2,\cdots,k, i = 0,1,\cdots,\left[\frac{N-m}{k}\right]$
      c. The length of x_k^m:

$$L_m(k) = \left\{\left(\sum_{i=1}^{\left[\frac{N-m}{k}\right]} |x(m+ik) - x(m+(i-1)\cdot k)|\right) \frac{N-1}{\left[\frac{N-m}{k}\right]\cdot k}\right\} \Big/ k$$

      d. Mean of $L_m(k)$: $\mathrm{L}(k) = \frac{1}{k}\sum_{m=1}^{k} L_m(k)$
      e. The slope obtained by fitting $\ln\left(\frac{1}{k}\right) \sim \ln\left(\mathrm{L}(k)\right)$ is the Higuchi FD

      k is 2 ~ 16
   (2) SFD:
      a. S（τ）=[z（x+τ）-z(x)2]=Cτ4-2D

      z(x+τ)-z(x)2 represents the arithmetic mean of variance; τ is an arbitrarily selected value of the data interval; Calculate the corresponding S(τ) for discrete signals of several scales τ versus fractal curve.
      b. Fit the slope of the log S(τ)~logτ to W
      c. SFD=4-W/2

The above two characteristic FD values are the FD mean values of 1400 RF time series in the ROI.

2. Frequency domain parameters:
   (1) Set each RF time series of length L in the ROI as {X(i): 1≤i≤L }
   (2) Perform Fourier transform on the RF time series to obtain the frequency spectrum X (k)
   (3) Calculate the average value $X_{ave}(k)$ of the spectrum X (k) at the same frequency in the ROI
   (4) Normalize $X_{ave}(k)$ to get the normalized spectrum: $\hat{X}_{ave}(k) = |X_{ave}(k)| \big/ \max(|X_{ave}(k)|)$
   (5) Perform straight-line fitting on the normalized spectrum to obtain the Slope, Intercept and Midbandfit.
   (6) Obtain S_1, S_2, S_3 and S_4 according to the following formulas:

$$S_1 = \sum_{k=1}^{L/8} \hat{X}_{ave}(k), S_2 = \sum_{k=L/8+1}^{L/4} \hat{X}_{ave}(k)$$

$$S_3 = \sum_{k=L/4+1}^{3L/8} \hat{X}_{ave}(k) \ , S_4 = \sum_{k=3L/8+1}^{L/2} \hat{X}_{ave}(k)$$

3. Time domain parameters:

(1) Kurtosis

Kurtosis describes the sensitivity of a time series to extreme values. The greater the time series changes, the greater the value of kurtosis.

$$\text{Kurtosis} = \frac{\frac{1}{n}\sum_{i=1}^{n}(x_i-\bar{x})^4}{(\frac{1}{n}\sum_{i=1}^{n}(x_i-\bar{x})^2)^2} - 3.0$$

$\{x_i: 1 \le i \le n\}$ is the RF time series, n and x are the length and mean of the RF time series, respectively.

(2) Peak

Peak describes the maximum fluctuation range of the time series. The peak value is defined as the mean value of the first L large amplitude absolute values of the RF time series

$$\text{Peak} = (1/L \sum_{i=1}^{L}|\mathrm{x}(i)|)$$

In this study, L is taken as 10, $\{\mathrm{x}(i): 1 \le i \le L\}$ represents the first L maximum values of the RF time series.

(3) Fuzzy entropy

Fuzzy entropy as a measure of the regularity of time series, has good robustness to noise.

a. Suppose the time series of N points is $\{\mathrm{x(i)}: 1 \le \mathrm{i} \le \mathrm{N}\}$

b. Regenerate a set of m-dimensional vectors in sequence order

$$x_i^m = \{x(i), x(i+1), \cdots, x(i+m-1)\} - x_0(i), i = 1,2,\cdots,N-m, x_0(i) = \frac{1}{m}\sum_{j=0}^{m-1} x(i+j)$$

c. d_{ij}^m is the distance between vector x_i^m and x_j^m

$$d_{ij}^m = \max_{k\in(0,m-1)}\{|x(i+k) - x_0(i) - (x(j+k) - x_0(j))|\}$$

$$i,j = 1,2,\cdots,N-m, j \neq i$$

d. Define the similarity of x_i^m and x_j^m:$D_{ij}^m = \exp(^{-(d_{ij}^m)^n}/_r)$

e. Define function:

$$\varphi^n(\mathrm{n},r) = \frac{1}{N-m}\sum_{i=1}^{N-m}(\frac{1}{N-m-1}\sum_{j=1,j\neq i}^{N-m} D_{ij}^m)$$

f. Repeat the steps e to d to reconstruct a set of m+1-dimensional new vectors according to the sequence order

g. $\varphi^{n+1}(\mathrm{n},r) = \frac{1}{N-m}\sum_{i=1}^{N-m}(\frac{1}{N-m-1}\sum_{j=1,j\neq i}^{N-m} D_{ij}^{m+1})$

h. Fuzzy enropy（m， n， r， N） $= \ln\varphi^{n}(n,r) - \ln\varphi^{n+1}(\mathrm{n},r)$

(4) Cross Zero Count and Cross Zero Std

The zero-crossing analysis method is not sensitive to interference and can quantify the details of the time series waveform structure. Compared with traditional frequency domain analysis, there will be more accurate results. The Cross Zero Count reflects the complexity of the time series, and the Cross Zero Std reflects the time series vibration Complexity.

The specific calculation steps are as follows:

a. Suppose the time series of N points is $\{\mathrm{x(i)}: 1 \leq \mathrm{i} \leq \mathrm{N}\}$

b. De-averaging: $\mathrm{y(i)} = \mathrm{x(i)} - \bar{x}$

c. Find the number of Cross Zero Counts in $\{\mathrm{y(i)}\}$, calculate the number of points between two adjacent Cross Zero Counts, and record it as

$d_1, d_2, \cdots, d_M$

d. Calculate the mean of the series $d_1, d_2, \cdots, d_M$

Cross Zero Count = $\frac{1}{M}\sum_{m=1}^{M} d_m$

e. Cross Zero Std = $\frac{1}{\mathrm{mZCI}}\sqrt{\frac{1}{M-1}\sum_{m=1}^{M}(d_m - \mathrm{mZCI})^2}$

Calculate fuzzy entropy, kurtosis, peak value, Cross Zero Count and Cross Zero Std for each RF time series in ROI in turn. Then calculate the mean value of the 1400 RF time series feature values in the ROI to obtain the time domain features of the lesion area.
